# Prostitution and Its Sanitary Management

**Published:** 1871-02

**Authors:** Edmund Andrews

**Affiliations:** Professor of Principles and Practice of Surgery in Chicago Medical College


CHICAGO

MEDICAL EXAMINER:

N. S. DAVIS, M.D., EDITOR.

F. H. DAVIS, ASSISTANT EDITOR.

VOL. XII. FEBRUARY, 1871. NO. 2

### Original Contributions.

### PROSTITUTION AND ITS SANITARY MANAGEMENT.

By EDMUND ANDREWS, M.D., Professor of Principles and Practice of Surgery in Chicago Medical College.

Prostitution is defined to be sexual congress for gain. Three years ago I published in THE EXAMINER my investigations into the results of the license system adopted in Europe for the regulation of this evil; showing its utter failure to diminish disease, and its positive effect to increase vice. My facts and deductions were received with incredulity by many, but, since that time, European writers have taken up the same topic, and been forced by facts and statistics to the same conclusions.

At the present time there is a renewed movement in favor of importing into our large cities, what is called the French system of regulation, and many very intelligent persons are anxious for its adoption in full French form, forgetful of the fact, that American reforms can only succeed when carried out on American plans, and not on worn out systems which have already proved worthless beyond the sea.

The facts and figures which I shall adduce, will show that the French plan is adapted, neither to the genius of our government, nor to the habits and character of our people. It was born of a public sentiment that neither hoped nor desired any diminution of the prostitution itself, but only such a limitation of its effects, as should enable men to wallow in its moral nastiness, without contracting physical disease. The American public sentiment is as different from this, as the north wind is from the breath of a cess-pool. We demand diminution of the vice, reform of the unfortunates, and limitation of the disease, and, we expect, in some good degree, to realize all of these objects together.

The usual arguments, on both sides, are these: First, on the side of the French scheme, it is said that prostitution has always existed, and that it is practically impossible to extinguish it by police regulations, and hence it is better to license it, and put it under sanitary control with the hope of limiting the spread of venereal diseases. It is further urged, that the patrons of prostitutes are not the only sufferers, but they marry innocent parties, and transmit to their wives and children the diseases which they contracted in the houses of infamy. Now, a weekly inspection of the prostitutes, and a consignment of all the diseased, each time, to a prison hospital until cured, might be expected to greatly diminish the venereal disorder among them, and thus make them much safer companions to their patrons, and diminish the amount of infection carried from them to innocent wives at home. It is hoped, also, by having the prostitutes under control, to abate their disorderly general behavior.

On the other side, it is replied, that the legalization of fornication is abhorrent to the moral sense of the community. If it is true, that it is impossible to entirely extinguish prostitution by police measures, the same is true of gambling, confidence operations, burglary, and murder. These have never been extinguished, but neither should they be licensed. It is feared, also, that the effect of giving prostitution a legal sanction, would be to throw around it a false respectability, which would greatly increase its influence, and by increasing its amount make the diseases fully as prevalent as before.

These arguments, both *pro* and *con*, are purely theoretical. We want the facts and the figures. What is the license system? What are its results? Does it diminish the disease in communities where it exists? Does it increase the prostitution? Let us seek for the answer.

In order, the better to arrive at some conclusion, as to what are the duties of American cities in respect to this evil, it will be well to take a brief survey of the measures which have failed in times past.

Numerous efforts have been made to suppress prostitution by the power of the law, but they have uniformly failed, and ended in either a tacit or an express toleration. The fact is, there are two classes of social offences in the world: one class, such as theft, burglary, and murder, is committed upon unwilling victims. In these cases, the sufferer, by his vigilance and his desire of safety of person and property, is the ally of the police, and does his best to help them prevent the crimes, or to punish the criminals; but, in prostitution, gambling, and drunkenness, both parties are in collusion, and do their best to shield themselves and each other from detection or interference, hence the police have no allies, and, in these offences, have always been obliged to tolerate misdemeanors of whose existence they were well aware, to an extent much greater than they do in theft and robbery. In these things, the police can only succeed in proportion as they are sustained by an increased moral purity, and intellectual advancement of the public.

Among the Roman emperors, Constantine the Great, Theodosius I, Theodosius II, and Justinian enacted severe measures against prostitution, but, as they ruled over cities whose corruption was so profound as to be almost inconceivable to modern minds, it is not likely that they met with any great success in their efforts at repression.

About the year 1254, St.Louis of France, after his return from Palestine, made two vigorous efforts to suppress the vice, but was finally forced to tolerate prostitutes in certain quarters of the cities. In 1560, an edict ordered the suppression of all the houses in France. The corrupt public sentiment gave no support to the law, and in Paris, great popular resistance was made; however, in about five years, the energy of the government succeeded in closing all the brothels in the city, but there sprang up such a number of private places of vice, that the evils of these secret dens, combined with the difficulty of maintaining a constant police tension against popular sentiment, caused a relaxation of the efforts, and a return to a sort of variable mixture of toleration and repression. (*Prostitution dans la ville de Paris, par Parent Duchatelet.*)

In Spain, the popular corruption became so great, that even the clergy and the convents were contaminated. The government attempted to stem the torrent by several severe prohibitory edicts, but in vain. Discouraged by bad success, toward the end of the 15th century a system of toleration and regulation was adopted, but, in 1623, there was so much dissatisfaction with that plan, that Philip IV again resorted to repression. In 1700, the government seemed to become again discouraged with that policy, and abandoned it, doing nothing more of any consequence until five years ago, when they again took up the toleration and regulation system.

In Berlin, prostitutes were tolerated in a do-nothing kind of way until the reformation, when repressive measures were attempted, but with such poor success, that after some variations, they were abandoned. The prostitutes were now tolerated, but required to live in a certain quarter of the city. This produced such a decline of the value of property in that vicinity, that real estate owners brought great pressure to bear on the government, and in 1844 a new suppression was attempted. After ten years of repression, during which it is claimed, that vice and disease increased, the present system of toleration and regulation was adopted, which, in its turn, is falling into some disrepute, from the utter inability of the police, by their own confession, to get more than a small fraction of those, whom they believe to be prostitutes, under registration and control. (*Westminster Review*, articles on prostitution, 1869–70.)

In England and the United States, no very decided policy was pursued until recently, the general plan being to have laws on the subject, but not to push them to any vigorous execution. In Philadelphia, however, the police at one time assailed the prostitutes with such energy, that at length there was not a single known brothel left in the city. To what extent this really diminished prostitution and its diseases, it is not easy to say, as no statistics were collected, but they met with an evil of another sort, which appears to have always cropped out when police efforts are more energetic than the morality of the community will sustain. The trouble referred to is this, the more crafty of the strumpets, driven from their usual haunts, boldly got recommendations of good character, and took positions as servants and nurses in respectable families, and thus carried on their business under cover, often by the connivance of the master of the house. The horror of respectable housekeepers who chanced to have sons growing to manhood, with such women in daily contact with them, may be easily conceived. This difficulty, combined with the fact that police energy can never be kept long straining at an aim much above the current morality of the community, broke down the plan, and caused a return to a sort of toleration.

In Chicago, no system of any kind has been regularly pursued. The police have never believed that they could eradicate the houses, and have felt obliged to give them a kind of half toleration. They have, however, made a practice of moderately harrassing them by "pulling the houses," that is, arresting, occasionally, all the inmates and fining them. There was, however, no serious expectation of breaking them up, as the women paid their fines and immediately resumed their business in the same places. During the administration of Mayor Wentworth, some ten years ago, these measures were pursued with more vigor than usual, and a disreputable street on the "Sands," entirely occupied by these creatures, was broken up and evacuated, thus removing a very disgraceful publicity of the vice.

The general result of all experiments at repression is this: In small towns of high moral tone, the combination of a virtuous and educated public sentiment, with judicious police action, is able to make a very encouraging headway against this class of offenders, but where there is a large class of sailors, immigrants, and vicious persons, in short, where public sentiment does not go hand-in-hand with all their measures, the police are left without allies, and can do almost nothing. It further appears, that when police action is at once severe and far ahead of the moral sentiment of the population, the prostitutes are driven into private families and other places as employés, and become, by contact with respectable young persons, new centres of corruption. We may, therefore, without hesitation, draw this conclusion. There is no wonderful quack remedy for the immediate cure of this evil. It must be attacked with broad plans and wide measures. It will be suppressed just in proportion as public virtue and intelligence grow among all classes, and lend their support and power to any police measures which may be adopted. Repression must be based on reform.

THE LICENSE SYSTEM.

The failure of repression having become evident, the idea sprung up of endeavoring to register, license, and regulate the prostitutes, and by placing them under official and medical control to eradicate their diseases.

On the continent of Europe, this plan has been adopted in Spain, Italy, France, Holland, Belgium, and Prussia. A similar measure has recently been introduced into some of the garrisoned towns in England, and, also, during the past year, into the city of St.Louis, in this country. The details vary, but the general plan is this:

1st. All prostitutes are tolerated on condition of registering their names with the police.

2d. They must live only where the police permit them.

3d. They must appear once or twice a week at certain designated places, where two physicians examine them, allowing the healthy to return to their abodes, and sending the diseased to a prison hospital until they are cured.

4th. They must pursue a quiet, orderly, unostentatious mode of life.

5th. Those not registering, or not conforming to the rules, are subject to fines and imprisonments.

In England, this plan was adopted, as it were, clandestinely. The popular sentiment being so far jealous of individual liberty, that it was not believed that the right to arrest and imprison these women in hospitals, without trial, would be granted if asked for openly, a law was got through in 1866, ostensibly to improve the sanitary condition of the army and navy. It granted power to the superintendents of police, in five naval towns, to arrest any woman in the place, or within fifteen miles of it, who should be complained of as believed to be a prostitute, and subject her to a forced medical examination. If she was found to be diseased, she was to be imprisoned in hospital, without trial, or any legal determination of the question whether she was really a prostitute. Soon after, the act was quietly amended so as to include all places where there was any naval station, garrison, fort, or battery, so that, without any public discussion or popular consent, and under cover of an act nominally intended to improve the sanitary condition of the army and navy, the people discovered that nearly the whole kingdom had been placed under a law, by which the malice or blundering of a superintendent of police could ruin the reputation of any woman in his district, without the possibility of protection or remedy. (*Westminster Review*, April, 1870.) This revelation excites considerable popular indignation.

The regulation system was adopted in St.Louis, (U. S.,) during the past year, and in form differs somewhat from the French plan, being apparently less perfect. It consists of an ordinance requiring the usual registry, etc., and dividing the city into six districts, each of which is under the care of one medical examiner. The latter has a salary of from $1200 to $2500 per annum, and goes alone to houses and apartments of the prostitutes, where he is directed to make inquiries, and, *if he thinks necessary*, physical examinations. He then gives such sanitary directions as he judges best, and orders any of the inmates to be removed to hospital, whose condition, in his opinion, requires it. There appear to be two mistakes here: 1st. As the physician is allowed his discretion about the physical examinations, and as he can save a vast amount of time and labor by omitting them, it may be expected that this part of the work will be very inefficiently done. 2d. The plan of sending young physicians alone to private interviews with young and handsome women of this character, often living semi-respectibly in their own apartments, will infallibly corrupt a large part of the examiners, and when the facts begin to come out, it will bring the characters of all of them into scandalous disrepute. The French avoid these objections by requiring the prostitutes to come to specified places for examination, where two physicians are in attendance together. They also endeavor to secure thoroughness by requiring the physical examination to be invariably made. The St. Louis establishments are taxed as follows: The keeper of the house $10 a month, and $1 a week for every girl in it, besides which the girls pay 50 cts. a week, each, so that every prostitute pays about $26 a year, and each keeper of a brothel about $300 on an average. The funds collected are applied to the hospital expenses.

RESULTS OF THE EFFORTS AT REGULATION.

The proposition to register and regulate what we find ourselves compelled in some form to tolerate, has, at first glance, such an air of practical common sense about it, that most persons are predisposed to hope more from the plan than experience justifies. The idea of compelling all the prostitutes to register, examining them weekly, and taking all the diseased ones under treatment, has such an appearance of completeness and practicability that one is predisposed to say, at once, that here is a plan capable of rooting out most of the venereal diseases that afflict the community. In this matter, however, as in many others, "'Tis distance lends enchantment to the view," and the theory is not sustained by the results.

The first essential condition of the compulsory license system, is a reasonably complete registry of the prostitutes, for, if the police do not succeed in enrolling their names, they will never get control of their persons, nor eradicate their diseases. As the first step, therefore, we must inquire into the success of

COMPULSORY REGISTRATION.

To those who have not investigated this subject in practical form, the idea of compelling the women to register seems very simple and easy. All that is necessary, they say, is to pass an ordinance to that effect, providing suitable penalties for those who refuse, and it will be done. The experience, however, of the most powerful and best organized police forces of the world, has confronted us, at the outset, with a dismal failure. Compelling women to do anything they do not wish to, seems to terminate about like Gov. Peter Stuyvesant's efforts to make the Dutch girls of New Amsterdam wear more petticoats. He was forced to desist enforcing his ordinance for fear they would at length leave them off altogether.

It is found on the continent of Europe, that the women resist and evade the registry to the utmost of their power, braving all the penalties, in such vast numbers, that the police is to a great extent powerless.

In Brussels, December 1, 1868, the registered women inside the walls of the city were 316, while, at the same time, those who escaped registry were estimated at 350. Of the registered women, 153, or nearly half, were diseased during the year, in spite of the sanitary measures. Outside the walls there resided great numbers more, who contrived to defeat the registration. (*Acton on Prostitution.*)

In Rotterdam, in eleven years, from 1857 to 1867, inclusive, the average number of registered prostitutes was 313, while the actual arrests of clandestine strumpets was 141, from which the large number of others, whom the police suspect but cannot convict, may be readily inferred. A physician in Rotterdam says the clandestines are very numerous, and that mothers lead on their daughters, and parents baffle the police by claiming and recovering their minor daughters, in order to profit by their clandestine prostitution. (Young clandestine girls will often bring in much higher profits than those who are known as public strumpets.)

At the Hague, in Holland, the commissary of police says the number of clandestines known to the police, but whom they cannot get under control, is "always increasing," and he is sure that the number not known to them is still greater.

A gentleman writing from there says, "many women, desirous to dress more finely, obtain the means to do it by prostitution, and the police know very well, that they cannot, and dare not, place the majority of such women under control."

Dr. John Webster, who wrote an account of prostitution in Naples, which has the same system of regulation as Paris, says, claiming to speak from the information of official documents, that more than half the registered prostitutes were found diseased, and that there were large numbers of clandestines. He remarks, "indeed, there, as in Paris and elsewhere, police laws do not diminish prostitution, nay, they even augment immorality."

In Paris, the utter inability of the police to compel any large proportion of the prostitutes to register, has, of late, been exciting public attention and discussion. M. Leon Lefort, Surgeon of the *Hopital du Midi*, with characteristic French faith in the omnipotence of government to abolish all the evils of society, comes out in a work advocating more vigorous efforts. He calls for the establishment of a special police force large enough to manage 50,000 women, with hospital facilities and a medical corps to correspond, and then demands, that all the loose women, not excepting even young girls living under the parental roof, be taken by the strong arm of the law, and placed in the public brothels. (*Lefort on the Prostitution of Paris in Relation to the Propagation of Venereal Diseases.*)

This project to provide for 50,000 of these women, compared with the fact that the police have been able to place under control less than 4000, is a startling confession of the failure of the system. M. Lecour, Chief of the *Bureau des Mœurs*, whose means of information are better than any one else's, estimates the number of Parisian prostitutes who escape registry at 30,-000, while there are only 3853 on the book of record. (*Westminster Quarterly Review*, from April, 1869, to April, 1870.)

In Berlin, in 1868, the number of registered women was 1650, while the number of those "suspected" was 13,306, and the

"suspected but more circumspect" were about 12,000. It appears, therefore, that in Paris the officers in charge of the matter are of the opinion that they have got less than one-seventh of the prostitutes under control, and in Berlin they have got less than one-fifteenth of those suspected. (*Westminster Quarterly Review*, Jan., 1870.)

Parent-Duchatelet, a former Chief of Police in Paris, makes the assertion that a review of the experience of the police, forces the conviction of the "uselessness of all measures taken by the administration to prevent prostitutes from inhabiting hired apartments (*les maisons garnies*) and prostituting themselves there just as in the public brothels." (See Duchatelet's *Prostitution dans la Ville de Paris.*)

The conclusion of this part of the subject is plainly this: The very first step in the regulation system of Europe, *viz.:* the compulsory registration, is a dismal failure. It is a very easy thing to say that every prostitute shall be registered or punished, and those who have not tried it, generally suppose that there will be no special difficulty in that part of the enterprise, but theories are sometimes erroneous. The stubborn fact is, that in defiance of authority, nine-tenths of these women will not register, and the police of Paris and Berlin, both far more despotic than public sentiment will ever permit ours to be, have found it impossible to compel them. Experience has brought to light the reasons of this unexpected result, of which the following are a part:

1st. There is, among the men who patronize such things, a preference for clandestine women, which, according to my observation as a physician, is strong enough to baffle police power. Men generally have some disgust at the idea of a woman, who is an openly recognized strumpet, whose vagina they know to be a common sewer for the evacuations of every beastly wretch who tenders the customary fee; but the clandestine woman is surrounded with all the charm of secrecy, with the appearance of decency, and, to some extent, even of womanly modesty. She lives retired and not known as a prostitute, generally having some reputable business as part of her subsistence, and her favors are often not given promiscuously, but only to some selected ones. Indeed, many young men go to their apartments under the impression that they have won favors of a person almost wholly virtuous. Every physician knows how general this idea is among a certain class of young men, even after they have caught disease from the supposed virtuous female. Now, the demand creates the supply, and this preference of the men, which is a part of human nature, always ensures that most of the loose women will be clandestines, for the simple reason that most of the demand and the pay is for such.

2d. This preference of the men is strengthened by the fact, that one who goes to a known house of prostitution, must run much risk of meeting, either there, or in entering and departing, some one who knows him and will divulge his visit, a danger which he avoids in calling upon a respectable clandestine.

3d. Experience and the testimony of the European police, proves, that the women themselves, for many reasons, revolt with all their power against being placed under control. In the first place, many are acting in the capacity of kept mistresses, and no police in the world ever pretends to register these. A large proportion of the rest have still some hold on respectability, and cherish, secretly, hopes of reform and reputable marriage, a hope which they not unfrequently realize. When they are arrested by the police, they are rudely torn from all these expectations of happiness, and plunged at once into an abyss of despair and infamy. They would be less than human if they did not resist and evade this horror by every effort and subterfuge within their power.

4th. Many young prostitutes are minors living with their parents, and, though debauched in morals, still in an attitude where there are hopes of reform and of marriage. Now, in such cases, as well as in those of older women who are quiet, modest in demeanor, and not generally known as prostitutes, with hopes of better things before them, the police magistrates naturally and properly hesitate to tear them from their shelter, and from their last hopes of virtue and reform, by consigning them to the position of open prostitutes; besides, the popular resentment excited by arresting this class, is such, that the police practically cannot do it.

5th. Most women utterly abhor the medical examinations of their persons, and the more so, as they must of necessity, from time to time be found diseased, and then they will be imprisoned in a hospital until their recovery, a period sometimes of many months. Hence the question is presented to them in this light, if they register, they are sure of being imprisoned every time they are diseased, whereas, if they evade the registry, and present the appearance of quiet, respectable women, with an apparent means of subsistence, they hope to escape imprisonment altogether, as well as the shame, exposure, and other horrors of being known as public whores.

With all this array of motives, it is no wonder that the registry is a failure, and we readily see that it is not any fault of the police that they cannot enforce measures which are opposed by the strongest instincts of human nature.

It may throw some light on the character, motives, and position of clandestine prostitutes, to give a few of the cases which have come under my notice as a professional man. I have altered the initials and unimportant details enough to avoid betraying professional confidence:

A. S.—Married—has been handsome. Her husband is a drunkard, and leaves her with but little means of subsistence. She ekes out her support by quietly receiving a few elderly men of means, who pay well for select favors.

M. M. G.—Married—costumer by trade. Takes subordinate parts in theatre. Member of a Protestant church. Has a family of children. Not handsome. Has a few appointments with widowers, etc. Sunk by degrees to a lower and lower level.

B. G.—Servant girl—Catholic in religion. A giddy, thoughtless girl, fond of display. Gets means to dress occasionally by submitting to men willing to pay.

L. D.—Married—English—husband a retail grocer. Has a family of young children to whom she appears devoted. Adds to the general income of the establishment, apparently with the connivance of her husband, by receiving sundry well-paying men.

B. S.—Quite a youthful-looking girl. Acted as mistress to a wealthy man, who furnished her with means and dress. Becoming dissatisfied with her paramour, she left him and took up with a small manufacturer—living in his house—passing as his niece, and acting as housekeeper. Mental distress at her position and prospects began to tell on her health, and she was seriously threatened by symptoms of pulmonary consumption. Learning that she had a respectable father living, who would be glad to receive her home, I advised her to leave her paramour, return to the parental roof, and by reform and good care endeavor to avoid the threatened consumption. She accordingly told her lover that she had resolved to leave him. He was surprised, and after a few minutes reflection told her that he could not do without her, and would marry her, and sending for a clergymen he immediately fulfilled his promise. Relieved of the mental horror inspired by her degraded condition and prospects, she became at once cheerful, recovered her health, spirits, and beauty, and made a happy and virtuous wife.

M. C.—Widow. Lived with a respectable married sister, the wife of a captain of a lake vessel. She made appointments with single gentlemen—going to their apartments, and not receiving them at her own room. As the years passed on she sunk lower, and finally went into a brothel.

A. A.—Dressmaker—unmarried—not handsome, but has a charming air of reserve and modesty. She apparently seeks no appointments, but yields to the solicitations of men of the higher class, and accepts such pecuniary recompense as they give her, each one being of the opinion that he has been almost the sole recipient of her favor.

R. L.—A widow in straightened circumstances. Took to receiving all sorts of men, and soon acquired the reputation of a common strumpet. Seems to have had very little sense of honor and decency, but became alarmed at the disturbances created by her conduct, and either reformed, or else became more secret in her vice. Retired to live a quiet life in the house of her brother.

These are fair samples of clandestine prostitutes, and in them may be seen some of the complex influences which make it certain, not only that they will not register as open strumpets until compelled, but, that in most instances, no police will think best to attempt the compulsion.

Experience shows, that the police are yearly losing ground in the matter of registry, and the prostitutes, in annually increased numbers, defy the law, and rush to supply the demand for clandestine paramours.

In Paris, where the effort of the police has been, so far as possible, to cause all the registered women to live in the public brothels, the result is, that while in 1857 there were 1976 women in the brothels; in 1867, when the population of the city had greatly increased, they had diminished to 1302. M. Lecour, the Chief of the *Bureau des Mœurs*, says, that the number of licensed houses in Paris and its suburbs in 1842, was 229, but in 1854, in spite of the exertions of the police and the growth of the city, they had diminished to 204, showing that the prostitutes were seeking clandestine abodes. The number of registered women fell off from the same cause from 4171, in the year 1841, to 3853, in the year 1867.

In Rotterdam, during the same ten years, the registered women diminished from 353 to 258, while the population increased materially. At the Hague, during the same ten years, the brothels diminished from 15 to 9, and the number of registered women from 130 to 82. (*Westminster Review*, January, 1870.)

It thus appears, that not only have the great majority of the women escaped the forced registry heretofore, but their skill in evasion increases, and the small fraction now on the books is yearly growing less.

FAILURE OF THE LICENSE SYSTEM TO DIMINISH DISEASE.

The great argument, and the only one of any value, used in favor of Americans copying the European system, is the assertion, that thereby we shall very greatly diminish venereal disease in the community, and, in fact, almost eradicate it, as vaccination has done to small pox. Here lies the pith of the whole matter. If this could be effected, the European plan would deserve, at least, an attentive consideration, to see if it were adapted to American circumstances.

The fact above shown, that the police of Europe only get control, according to their own testimony, of a small fraction of the prostitutes, is adapted to sober our hopes of eradicating syphilis by copying their method. The best source of information is the amount of disease among the soldiers, before and after the adoption of this plan. In England, the "Contagious Diseases Act" of 1866, was applied to five districts, for the express purpose of improving the health of the army and navy, with the following results. (*Westminster Review*, Jan., 1870.)

TABLE SHOWING THE NUMBER OF ADMISSIONS INTO MILITARY HOSPITALS PER 1000, OF MEAN STRENGTH BEFORE AND AFTER THE ACT:

| Districts, | 1865 | 1866 | 1867 | 1868 | Act went in effect. |
|---|---|---|---|---|---|
| Devonport and Plymouth, | 360 | 317 | 312 | 280 | Oct. 10, 1866. |
| Portsmouth, | 329 | 359 | 378 | 348 | " 8, " |
| Chatham and Sheerness, | 292 | 326 | 277 | 275 | Nov. 6, " |
| Woolwich, | 204 | 219 | 255 | 191 | " 6, " |
| Aldershot, | 302 | 233 | 261 | 237 | Apl. 12, 1867. |

From this it appears, that though in the first two districts, there was, in 1867, a slight diminution of disease, which has been much extolled by writers as due to the operation of the Act, yet, in the three others, there was an increase more than sufficient to balance it, so that, for two years there was, on the whole, an increase of disease, which, however, diminished again subsequently. In this case, the Act had, probably, little to do with the increase or diminution; for, statistics show, that owing to better management and improved habits of life, the health of the army had been, on the whole, improving for years, and the figures happen to show that this improvement was more rapid before the passage of the License Act than after. The following table from a writer in the *Pall Mall Gazette*, March 3, 1870, makes this evident by comparison with the previous table:

AVERAGE RATIO PER 1000 OF MEAN STRENGH OF ADMISSION TO MILITARY HOSPITALS:

| Years, | 1860 | 1861 | 1862 | 1863 | 1864 | 1865 |
|---|---|---|---|---|---|---|
| Ratio, | 421.20 | 408.60 | 361.40 | 363.40 | 296. | 297.40 |

Or, in brief, for five years before the Act, there was an annual diminution of 21 admissions to hospital per 1000 of mean strength, while, for the two years after the Act, the diminution was only 12 per 1000, annually.

It appears, therefore, that the Act has not rendered the five districts to which it applied any better for the army. The operation of the Act on the British army in Bengal was perfectly similar, admissions to hospital under it having increased from 166 to 199 per 1000 of mean strength.

It has been claimed, that the French armies were made much more free from venereal diseases than the British army by the license system, and figures of the relative numbers of venereal cases admitted to hospitals are quoted from the military reports to sustain the idea; but the comparison is worthless, for this reason, in the French army the venereal cases are nearly all treated, as the phrase goes, "in quarters," and do not appear on hospital record, while in the British service they all go to the hospital, and stand recorded on the books. Hence the official registers of the two nations furnish no means of comparing the amount of venereal disease in their armies. In the army of Holland the same results are seen. After the license system was put in operation there, the venereal diseases among the soldiers diminished in some towns and increased in others, but the average result was an increase. Before the adoption of the plan, there were, in 15,913 soldiers, 1786 venereal cases, equal to 11.2 per cent., but since the act attempting forcible regulation, the venereal cases have increased to 13.3 per cent. (Statistics published by Dr. Huet, 1st physician to hospital at Amsterdam.)

In 1867, while traveling in Europe, I gathered statistics from non-licensed cities, to see how the ratio of venereal diseases to all kinds of cases in the civil hospitals, compared with the ratio in similar hospitals under the license system of Paris. The following is a brief summary of results:

TABLE SHOWING THE PROPORTION OF VENEREAL PATIENTS TO ALL KINDS TREATED IN CIVIL HOSPITALS WHERE NO LICENSE SYSTEM EXISTS:

| Cities, | Venereal Cases, | Cases of all kinds, |
|---|---|---|
| Chicago, | 580 | 4,147 |
| Philadelphia, | 1,127 | 12,292 |
| New York, | 1,833 | 23,314 |
| Liverpool, | 2,073 | 63,074* |
| Manchester, | 3,500 | 75,000* |
| London, | 3,357 | 31,264 |
| Totals, | 12,470 | 209,091 |

* The figures from Manchester and Liverpool appear large, because the out-patients were included; in the other cities there is no record of out-patients.

Or, one venereal case to about 16¾ of all kinds. In Paris, the published reports of the Prefect of Public Assistance, show as follows:

Venereal cases, ------------------------ 5,276,
Cases of all kinds, ---------------------84,267.

Or, one venereal case to about 16 of all kinds. I should remark, that had I included the Saint Lazare, which is the prison hospital for prostitutes, the French statistics would have appeared still worse, but I excluded that, because there is no similar thing in the other cities. As it is, venereal diseases, in spite of the supposed efficacy of the compulsory regulation, appear to be five or six per cent. more abundant than in cities which have no such system.

It is evident, therefore, that the European system of license and compulsory regulation has not made the slightest perceptible progress in eradicating, or even abating disease from the community, so that, in respect to the main object of its establisment, it is a costly and damaging failure.

How is this surprising result to be accounted for in the face of the fact, that the police do make a very marked diminution of venereal cases among the few prostitutes whom they succeed in getting under control?

The truth probably lies just here. In the first place, the number put under control, is only a small fraction of the licentious women in any city, so small that the improvement thus made amounts to but little, and secondly, the fact that the police are supposed to keep the strumpets free from disease, acts as a magnificently delusive advertisement to all the young men in the city, leading them to suppose that licentiousness has now become almost safe. They, therefore, rush into indulgence with greatly increased frequency, and find, to their cost, that syphilis is fully as easily found as before. In other words, the license system increases the patronage of vice more than it diminishes the disease, and, by multiplying the number of exposures, more than makes up the diminished risk of each particular act.

As a professional man I have been compelled to laugh at the frequent instances where young Americans have, with infinite gullibility, cohabited with loose women in Paris, because they supposed it safe there, but were utterly astounded afterwards to find they had contracted syphilis or gonorrhœa.

THE LICENSE SYSTEM INCREASES PROSTITUTION.

If the plan of license and forced registration, while making little or no impression on the amount of disease, actually increases prostitution in the community, American public sentiment will condemn it to oblivion. In 1867, I gathered from various sources, at some expense and much labor, the means of making a comparison between the number of prostitutes known to the police in the non-licensed cities, and those registered in the licensed ones. This method of comparison of the morals of the two classes of cities, does no injustice to the licensed portion, because the number of prostitutes practically known to the police, is always greater than the number they are able, as a matter of fact, to register.

TABLE SHOWING THE PROPORTION OF REGISTERED PROSTITUTES TO THE POPULATION IN LICENSED CITIES:

| | | | | | |
|---|---|---|---|---|---|
| Paris, | 1 | prostitute | to | 281 | inhabitants. |
| Brussels, | 1 | " | " | 435 | " |
| Berlin, | 1 | " | " | 437 | " |
| Copenhagen, | 1 | " | " | 564 | " |
| Hamburg, | 1 | " | " | 359 | " |

| | | | | | |
|---|---|---|---|---|---|
| La Haye, | 1 | prostitute | to | 750 | inhabitants, |
| Rotterdam, | 1 | " | " | 267 | " |
| Amsterdam, | 1 | " | " | 286 | " |
| Turin, | 1 | " | " | 193 | " |
| Bordeaux, | 1 | " | " | 270 | " |
| Brest, | 1 | " | " | 159 | " |
| Lyons, | 1 | " | " | 424 | " |
| Marseilles, | 1 | " | " | 286 | " |
| Nantz, | 1 | " | " | 378 | " |
| Strasbourg, | 1 | " | " | 251 | " |
| Algiers, | 1 | " | " | 102 | " |
| Average, | 1 | " | " | 342 | " |

TABLE SHOWING PROPORTION OF PROSTITUTES, KNOWN TO THE POLICE, TO THE POPULATION IN CITIES NOT LICENSED:

| | | | | | |
|---|---|---|---|---|---|
| Chicago, (1867), | 1 | prostitute | to | 230 | inhabitants. |
| New York and suburbs, | 1 | " | " | 518 | " |
| London and suburbs, | 1 | " | " | 544 | " |
| English great seaports, Liverpool, Bristol, & Plymouth, | 1 | " | " | 193 | " |
| English pleasure towns, Brighton, Bath, etc., etc., | 1 | " | " | 244 | " |
| English towns dependent on agricultural districts, as Ipswich, etc., | 1 | " | " | 309 | " |
| Seats of English cotton manufactures, as Manchester, etc., | 1 | " | " | 557 | " |
| Seats of English mixed manufactures, as Norwich, etc., | 1 | " | " | 478 | " |
| Seats of English woollen manufactures, as Leeds, etc., | 1 | " | " | 654 | " |
| Seats of Eng. Iron manufactures, as Birmingham, Sheffield, etc., | 1 | " | " | 709 | " |
| Glasgow, | 1 | " | " | 394 | " |
| Madrid, (before 1865), | 1 | " | " | 270 | " |
| Average, | 1 | " | " | 425 | " |

It appears, therefore, that the proportion of registered prostitutes to the population, is 24 per cent. greater in cities adopting the license system, than in those not adopting it. With this evidence before us of the demoralizing effect of the European system, we must certainly hesitate about copying it, and choose rather some more hopeful method.

There are two forces which act in the matter of prostitution. One of these is the police power, and the other, the force of society. Each of these has its proper field of effort, and may have its honorable victories.

Considering the police power first, it seems to me that the following, must of necessity, be an outline of its course. First, with regard to

TOLERATION.

This is a settled matter. In Chicago, as elsewhere, the police do not feel able, in the present state of public morals, to break up or materially diminish prostitution. They already tolerate it. The irregular system of "pulling the houses," before described, may have been a slight discouragement to prostitution, by rendering it a trifle more expensive, and by keeping some men away from night visits, lest the house should happen to be "pulled" while they were there, but, practically, it amounted to nothing. Moreover, it entailed a serious evil of this sort. It tended to corrupt the police, by rendering it possible for employés of the force to levy black-mail, under threat that they would use their influence to cause the houses to be "pulled." The system has proved, therefore, not only inefficient, but mischievous. Impressed by these facts, the Board of Police, have of late, discontinued the practice, and ordered that the "pulling" shall be exclusively reserved for houses whose inmates are offensive and disorderly in their general behavior. Toleration, therefore, in some form or other, is inevitable, simply because there is so much licentiousness among men that law cannot restrain it. At some future age this may be different, if morality and intelligence shall increase as much in the future as they have in the past, for, in spite of all croakers, there is much less licentiousness now than there was five hundred years ago; but at present, toleration, as a matter of fact, is established and inevitable, just as completely as it is in regard to gambling, which is immoral and illegal, but tolerated, because at present we cannot suppress it.

THE FORM OF TOLERATION.

American public sentiment is strong in its feeling, that any toleration granted, shall not assume an aspect of public sanction or approval of vice, and this feeling is not only strong, but eminently proper. We can readily see, that in various offences, we may find it best, to a certain extent, to remit the punishment, but never to withdraw our disapproval of the offence.

The English, with their characteristic bluntness, have committed an error in this respect in the execution of the Contagious Diseases Act of 1866. They give formal licenses to the tolerated houses, and regular certificates of health to the strumpets at each examination, which the latter can show to their patrons as an encouragement. The French, on the other hand, with their usual regard for the decency of public appearances, put their permissions in such a form as to recognize the offence, and they furnish the women with no certificate of health, but only a card memorandum of the date of the last medical examination.

If it is necessary to allow the prostitutes any permission, other than a verbal one, recorded on the books of the police, it should be in a form which recognizes the offensiveness of their profession, but grants them an exemption from the proper legal penalties during orderly behavior, or until further notice. A better attention to the decency of public acts than the English lawgivers have shown, will prevent much opposition on the part of some classes of society.

REGISTRY.

The police need for their purposes a list of these women, but we have seen above, that a compulsory registry is always a failure, the police of Paris, enrolling only 3853 out of about 30,000, and those of Berlin only 1650 out of 26,956 of those suspected. The reasons why the women resist the registry we have already dwelt upon, but, perhaps, the following ought to be added: The great mass of actual prostitutes, are not like the lowest class, debauched out of all shame, and bereft of hope of better things. They have friends whom they love, and who often do not know their errors. They have family connections and some relics of a good reputation, as well as their liberty to preserve. An European writer remarks thus: "For all these women registration at the *Bureau des Mœurs* means open social degradation; it sets upon them the mark of infamy; it compels them to commit themselves to a life of prostitution as a condition of continuing to exist, whereas, before they were but hovering on the brink of it, and still had it in their power to turn back; it means loss of valued acquaintances and of long cherished friends, and, worst of all, it means, also, but too often to be cast off by relatives, to be disowned and repudiated by father and mother, and thus virtually to be forbidden ever again to visit the beloved home of childhood and youth. An unregistered woman who has 'fallen,' or who has been tempted by any of the many reasons which impel women to prostitution, to prostitute herself temporarily, has it in her power to recover herself, and to resume her ordinary position in the society in which she moves, if, meanwhile, she discretly keeps her own counsel, as she is likely to do; but the difficulty of recovery after registration is increased a thousand fold."

For these reasons, a forced registry, whose immediate consequence is to place the victim under medical and police observation and control, not only is an unavoidable failure, but, in regard to the majority of these women, ought to be so. Any system which could successfully drag them all from the remnants of their virtue, and crush out the last of their hopes of better things, by launching them beyond recovery, into open prostitution, would petrify the civilized world with horror the moment it was put into execution. It seems to me, therefore, that there is no other way for the police to possess themselves of a useful register, but to make a classified secret list from the results of information gathered by the various ordinary means within their reach. In respect to open and confessed prostitutes, there would be no difficulty in obtaining the information directly, but with clandestines, even the all-powerful police of

Berlin, with all their laws, is obliged to proceed on this indirect plan. An absolutely complete forced registry might have some advantages, but compulsory measures which the best organized police bodies of Europe cannot execute, will be found equally impossible to the police of Chicago. A private list thus gathered and kept by the Chief of Police, would be free from the evils of the compulsory registration, because unaccompanied by the risk of exposure from medical inspection and hospital imprisonment.

TREATMENT.

This is the great end sought by means of the forced registry and the compulsory placing of the prostitutes under police control. There is, abroad, a very exaggerated idea of what can be accomplished by these means. In the first place, the physicians cannot proceed a step beyond the list in the forced register, and as six-sevenths of the women, at least, are found to escape the registry, they escape, also, the hated medical inspection and hospital imprisonment. But there is a most michievous error abroad as to what medical men can accomplish, even on those who are under control. The general supposition is, that when a physician has examined a woman and found no disease visible, she is entirely safe to her paramours. This is a fatal blunder, as many a man has found to his cost. The following facts, well known to professional men, but not so much so to non-professional readers, will explain my meaning:

There are three venereal diseases, gonorrhea, soft chancre, and hard chancre. The first two are purely local diseases, and do not produce any injury to the general constitution; the last is essentially a constitutional, as well as a local disease, and is the dreaded syphilis, which may break out anew after years of dormancy, and may be transmitted to offspring by inheritance. This last disease is the only one sufficiently important to demand public sanitary management. Now, the first two diseases may be experienced over and over again by the same person, but still individuals acquire a sort of partial insusceptibility to them, so that a practised prostitute will receive the poison from some diseased man, and carry it in the folds of her passages for days, without taking the disease herself. In this way a woman, who is herself perfectly healthy, and has nothing about her which a physician can discover to be wrong, may give disease to twenty men.

There is a peculiarity about the last disease, called hard chancre and constitutional syphilis, which places the matter in a still stronger light. This, the only really dangerous one of the diseases, like measles and small pox, can usually be had only once by the patient. The prostitute usually gets the disorder reasonably early in her course, and thus becomes incapable of it thereafter. So far as that disease is concerned, therefore, she can carry the poison with impunity to herself, but woe to all the men who copulate with her, if any diseased man has been before them within a few days. In this case the physician is utterly powerless. He examines her passages by a good light, it may be, and with the most conscientious care, and finds nothing in her condition which he can see to be wrong, and yet within that very hour, some patron may receive from her a disease which shall cause his death. In Paris, attempts are made to abate this danger by ordering the woman to syringe out her vagina after each copulation. This, if carried out, would help a little, but there are two difficulties here:

1st. The poison by the copulation is well incorporated with the secretions in all the deep folds and recesses of the vagina, and would not be effectually removed by any syringing which the woman will be likely to carry out.

2d. There is no way to compel the prostitute to do it, nor to detect her if she does not. There would be no possibility of getting it attended to, so as to be of any use, unless a couple of policemen were detailed to wait on her constantly, and syringe her out by force, for two hours continuously after each cohabitation.

A prostitute who has been with a syphilitic man, though she remain perfectly healthy, has her vagina saturated with the man's poison, and remains for several days as dangerous as though she had syphilis herself. Probably four-fifths of all the venereal cases in men, are derived, not directly from the woman's own poison, but from the virus wrought into her vaginal mucus by diseased men. By the same process, the poison is constantly renewed and kept on hand for the accommodation of her successive customers. In view of these well-known facts, it is a matter of utter astonishment, that any surgeon, or even any man of uneducated common sense, should suppose that a medical examination can give the least security to cohabitation with prostitutes.

These are among the reasons why medical inspection has proved an utter failure in Europe, so far as diminishing the disease in the community is concerned. There would be no possibility of checking the disease by such methods, unless the men as well as the women were examined, and all prostitution prevented, except where both parties were proved to be healthy. Now, I submit the question, whether it is advisable for the community to adopt a costly system, which, while it affects no diminution of general disease, acts as a delusive advertisement to lead men to suppose that the chambers of prostitution have at last become almost safe resorts.

PLAN FOR ACTION.

Though we have been baffled hitherto, it by no means follows that we are to fold our hands and do nothing. Although the hope of absolutely eradicating venereal disease will never be realized until prostitution itself ceases, yet we can do much in a sound, common sense manner to diminish its prevalence. There are only two things which will have any real effect in this direction, and they are these:

1st. Free treatment in hospitals for the prostitutes and all other venereal patients. Their residence in hospital should not be a compulsory imprisonment, which they will always resist and evade, but a voluntary thing to which they are to be led by kindly invitation, and by freedom from expense. In this way more prostitutes can be drawn into hospital than ever can be gotten there by force.

2d. A new plan of legal management by which police shall move on side by side with the lovers of humanity and religion in a grand movement of regulation and reform.

The police alone can do nothing. Society must go hand-in-hand with them, and then a work will be done of which the nation may well be proud. We shall presently show, that on the part of society, this work has already commenced, and shows such elements of power and success as promise the best results for the future.

Let us consider these two points in their proper order; and, first,

HOSPITAL ARRANGEMENTS.

In this matter the following plan seems to me feasible: Let an agreement be made with every hospital in the city to receive all women affected with venereal diseases who come with a permit from the proper officer. The woman is to be cheerfully welcomed, kindly treated, fed, lodged, and cured, free of expense, and the hospital is to be reimbursed from a fund derived from fines on disorderly persons and houses. If this is done, and a moderate police pressure be brought to bear on the more open houses of prostitution, to send up promptly all diseased cases, it will be found that more women, by far, can be made to avail themselves of it, than by a compulsory examination, and hospital imprisonment like that practised at the Saint Lazare in Paris. I advocate the distribution of women in ordinary hospitals, instead of building a separate one, for these reasons: A separate hospital for this purpose would be considered as purely an asylum for infamous characters, and attendance on it equivalent to a confession of open prostitution. The clandestine women would therefore avoid it, as they do all other forms of publicity, and, as they constitute the great majority of the female propagators of disease, the plan would defeat itself. Another thing is worthy of consideration. Most of the hospitals are under the care of religious bodies. Now, both the Protestants and the Roman Catholics have had in progress for years some very successful efforts for reforming these unfortunates, and they have already permanently restored to respectability nearly one thousand of them in this city. This work has been done silently, but it is of the most noble and successful character. Many prostitutes would, from preference, enter the denominational hospitals, and thus furnish a most admirable opportunity for the reformatory associations to bring them under their influence, and not a few would thus be cured, both morally and physically.

The following is a list of the hospitals which probably might be made available; possibly others, also, exist which do not occur to my memory at this moment:

On the South Side, we have the Hospital of the Sisters of Mercy, (Roman Catholic), and St.Luke's Hospital, (Protestant), and the County Hospital; on the North Side, there is the Hospital for Women, not denominational, but practically controlled by Protestants, the Hospital of the Sisters of Charity, and the Jewish Hospital.

Some of these institutions at present hesitate to receive this class of patients, but, if it were once understood that the desire of the authorities is, to go hand-in-hand with the lovers of religion and humanity, and by a united effort do what we can, both to limit disease and lift up the unfortunate, every hospital in the city would open its doors, and every religious denomination would applaud and sustain the plan.

To treat the women alone, however, with the hope of removing syphilis from the world, and leave the men diseased, is as hopeless as to draw out the water from one arm of a syphon, when the other is immersed in an exhaustless reservoir. Each of these hospitals should receive male patients at a free dispensary, daily, and treat them thoroughly; receiving from the fund before mentioned, a slight compensation to cover the expenses.

At present we have the following free dispensaries, most, or all of which could be made available:

On the South Side, the Davis Free Dispensary, kept at Mercy Hospital, the Dispensary at St.Luke's Hospital, and that at the County Hospital. On the North Side, there is the Dispensary at the Hospital for Women, (which would avail for female out-patients), and the General Dispensary at Rush Medical College; probably, also, the Sisters of Charity Hospital, the Brothers' Hospital, and, perhaps, the Jewish Hospital would open dispensary rooms, if desired. On the West Side, there is the Brainard Dispensary, which has been established several years, and would do good service. By these measures much would be gained, in fact, all that it is possible to do, until, by the growth of virtue and intelligence, and the diffusion of the knowledge of the dangers of prostitution to both parties, humanity shall be raised to a higher level, both of purity and of prudence.

### THE REFORM OF PROSTITUTES.

Some curious misconceptions are prevalent on this subject. Among them is the notion that women in good society have no pity or help to offer to the outcasts of their own sex, but do their utmost to trample them into hopeless infamy. Now, it happens that the only valuable work that has ever been done in this field, has been accomplished by women. In the same flippant way, it is charged that the religious portion of the community totally scorn and neglect them. Now, it happens, also, that a great work has been silently going on for years, and that it is carried on almost exclusively by religious women, who, in this city, have already permanently reformed nearly one thousand prostitutes. They have done it, too, with such modesty and silence, that the community is almost unconscious that anything has been attempted.

Two hundred and twenty years ago, Jean Eudes, of Caen, in France, took up this matter, and founded an order of nuns called the Sisters of the Good Shepherd, whose entire lives were devoted to the reform of prostitutes, and the education and rescue of little girls, daughters of abandoned parents, or orphans on the road to ruin. They have now 113 establishments, of which 21 are in America. The prostitutes, when they first enter these houses, are received into a class called penitents, and are kept actively occupied in such a round of labor, religious exercises, singing, etc., as to leave them little leisure for gloomy reflection, while, at the same time, their needle-work, etc., pays for their subsistence. Those who continue steadfast two years, and desire to live permanently in the house, enter into a sort of religious order, called Magdalens. The others are sheltered until employment or homes can be found for them.

Great numbers are permanently reformed, though, of course, many others backslide.

The entire order now has under its charge about 7000 Magdalens, and has in its schools about 8500 little girls, who were taken out of the high road to ruin.

The House of the Good Shepherd in Chicago was established twelve years ago. It has 14 Magdalens, and 42 young rescued girls in school, besides a variable class of penitents. This house has already reformed nearly 500 dissolute women in Chicago. The experience of the order, is, that of the women who come to them voluntarily, more than half are successfully and permanently reformed, but where they are sent there against their will (minors are sometimes sent by parents) they are self-willed and angry, and rarely do well, yet, after being released, some of them return voluntarily, and become permanently reformed. There is a good deal of human nature in this fact, which may be profitably studied by those, who think it would be easy for our police to hunt up and subject to compulsory regulation some thousands of such women.

The Erring Woman's Refuge for Reform in Chicago, has been established about eight years. It is managed by an association of ladies from Protestant churches. It has hitherto received about 75 inmates every year, two-thirds of whom are professional prostitutes, and the rest non-professional women, who have been seduced and thrown upon the world. The non-professional ones are nearly all saved to virtuous lives, and about half the professional ones. They have already permanently reformed, in the eight years of their operations, about 300 habitual prostitutes, besides saving about 200 seduced women from that infamy. Many of the women are known to be happily married, some of them in the upper class of society. The plan pursued is purely one of protection, kindness, and encouragement. The women are taught literature and music, and whatever else they need: employment is sought for them, and, in case of women seduced and abandoned when pregnant, measures are taken to compel the seducer to, either marry the woman, or provide her suitable pecuniary supplies. Similar

Protestant institutions are in operation in all the large cities.

These two reform establishments, receive by law, a dividend of all the fines levied on prostitutes and disorderly houses, which enables them to greatly increase their usefulness.

THE DUTY OF SOCIETY.

The day is gone by, when prostitutes were considered hopeless characters. The investigations of the police of New York and of Paris has developed the fact, that vast numbers of them annually abandon the business of their own accord, and it is known that thousands are rescued by the benevolent. *Prostitutes are reformable*, and one of the duties devolving on us is to give the efforts in that direction a greater vigor and power, and a more liberal pecuniary support. At present there are three reformatory institutions in Chicago. One, the Washingtonian Home, is for inebriety, and receives ten per cent. of the license fees collected by the city. The other two are the Protestant and the Roman Catholic prostitute asylums. The two latter receive a percentage of the fines inflicted upon houses of prostitution. There is, however, an oversight in the law, by which the income thus derived is rendered a small thing. The vast majority of all the offending prostitutes and brothel keepers are fined as simply "disorderly," and not as prostitutes, etc., and by the wording of the law fines for "disorderly conduct" cannot be paid to the asylums. We need a new act to remedy this deficiency.

Commissioner T. B. Brown, President of the Chicago Board of Police, makes the following suggestion:

The great majority of the fines inflicted for misdemeanors arise from drunkenness and prostitution; now, let the fines levied on vice go for the reform of the vicious. Let the Legislature be asked for a general law, by which all fines levied in such cases, shall, after deducting court expenses, go into a special fund for the support of reformatory and curative measures. Let the hospitals have a certain allowance, and, as to the rest, either let the inebriate asylum receive its present per cent., and also all the fines levied for violation of license laws, and for drunkenness; and the asylums for the reform of fallen women all the fines arising out of other misdemeanors (after deducting court expenses) or, else put the whole into one fund and divide it equally between the two classes, that is, half to the inebriate, and half to the prostitute asylums. In this way, police power, humanity, and religion, would all work harmoniously for the maintenance of order, the abatement of disease, and the elevation of mankind.

Finally, systematic efforts should be made through the press, and even the pulpit, to warn the young and ignorant, not only of the moral, but of the physical dangers of licentiousness. Especial pains should be taken to spread the knowledge of the fact, that women, not themselves diseased, are common-carriers of poison, and that there is no possibility by medical, or any other means, of rendering cohabitation with a prostitute safe.

To sum up all, we find that

1st. The European compulsory registry only enrolls a small fraction of the women.

2d. The system of forced medical examinations, with attempts to consign the diseased to hospital prison, totally fails to abate the prevalence of disease.

3d. It is better for us not to copy European failures, but to develop our own system.

4th. This system should consist, on the part of the police, in a strictly tacit toleration of the orderly prostitutes, a private classified registry, free hospital assistance for the diseased, and fines and imprisonments for the disorderly.

5th. On the part of society, there should be an extension of the present efforts to reform the fallen, and to rescue the young candidates for shame. Measures should also be taken, through the pulpit and the press, to warn the unwary of the physical as well as the moral dangers of licentiousness, and of the inefficiency of all known measures to render prostitution safe.

Note.—The reader desiring to investigate this subject will find the following to be the principal sources of published information:

1. *History of Prostitution*, by W. W. Sanger, M.D., New York.

2. *Prostitution dans la ville de Paris*, par Parent Duchatalet, complétée par M. M. Trebuchet et Poirat-Duval, Paris.

3. *Prostitution Considered in its Moral, Social, and Sanitary Aspects*, by William Acton, M.C.R.S.

4. *Eleventh Report of the Medical Officer of the British Privy Council.*

5. *Prostitution dans les Grandes Villes*, par le Docteur J. Jeannel.

6. *Prostitution and the License System of Europe*, in the CHICAGO MEDICAL EXAMINER, 1867: by Edmund Andrews, M.D., Professor of Surgery in Chicago Medical College.

7. *Report from the Select Committee of the House of Lords on the Contagious Diseases Act.* Session 1867–8.

8. *Histoire de la Prostitution*, par Pierre Dufour.

9. *Magdalenism: Causes and Consequences of Prostitution in Edinburgh*, by William Tait, Surgeon.

10. *Articles on Prostitution in the Westminster Quarterly Review*, July, 1850; April, July, and October, 1869; and January and April, 1870.

11. *Report of the sub-Committee of the Association for Promoting the Extension of the Contagious Diseases Act.* July, 1869, London.

12. *Publication of the National anti-Contagious Diseases Act Extension Association, including Papers by the* Rt. Hon. W. E. Gladstone, Prof. Newman, C. B. Taylor, and others.

13. *The Remedy Worse than the Disease*, published by the Society for the Rescue of Young Women and Children, London.

